# Liquid Biopsy in Glioblastoma

**DOI:** 10.3390/cancers14143394

**Published:** 2022-07-13

**Authors:** Lorian Ronvaux, Matteo Riva, An Coosemans, Marielle Herzog, Guillaume Rommelaere, Nathalie Donis, Lionel D’Hondt, Jonathan Douxfils

**Affiliations:** 1 Department of Pharmacy, Namur Research Institute for Life Sciences, University of Namur, 5000 Namur, Belgium; lorian.ronvaux@unamur.be; 2 Belgian Volition SRL, 5032 Isnes, Belgium; m.herzog@volition.com (M.H.); g.rommelaere@volition.com (G.R.); 3 Department of Neurosurgery, CHU UCL Namur Site Godinne, Université Catholique de Louvain, 5530 Yvoir, Belgium; matteo.riva@kuleuven.be; 4 Laboratory of Tumor Immunology and Immunotherapy, Department of Oncology, Leuven Cancer Institute, KU Leuven, 3000 Leuven, Belgium; an.coosemans@kuleuven.be; 5 Qualiblood s.a., Research and Development Department, 5000 Namur, Belgium; nathalie.donis@qualiblood.eu; 6 Department of Oncology, CHU UCL Namur Site Godinne, Université Catholique de Louvain, 5530 Yvoir, Belgium; lionel.dhondt@chuuclnamur.uclouvain.be

**Keywords:** glioblastoma, diagnosis, follow-up, biomarkers, circulating tumor DNA, circulating microRNAs, circulating tumor cells, extracellular vesicles, circulating nucleosomes

## Abstract

**Simple Summary:**

Glioblastoma is the most common and malignant primary brain tumor. Despite intensive research for new treatments, the survival of patients has not significantly improved in recent decades. Currently, glioblastoma is mainly diagnosed by neuroimaging techniques followed by histopathological and molecular analysis of the resected or biopsied tissue. Both imaging and tissue-based methods have, despite their advantages, some important limitations highlighting the necessity for alternative techniques such as liquid biopsy. It appears as an attractive and non-invasive alternative to support the diagnosis and the follow-up of patients with glioblastoma and to identify early recurrence. Liquid biopsy, primarily through blood tests, involves the detection and quantification of tumoral content released by tumors into the biofluids. The aim of the present review is to discuss the biological bases, the advantages, and the disadvantages of the most important circulating biomarkers so far proposed for glioblastoma.

**Abstract:**

Glioblastoma (GBM) is the most common and aggressive primary brain tumor. Despite recent advances in therapy modalities, the overall survival of GBM patients remains poor. GBM diagnosis relies on neuroimaging techniques. However, confirmation via histopathological and molecular analysis is necessary. Given the intrinsic limitations of such techniques, liquid biopsy (mainly via blood samples) emerged as a non-invasive and easy-to-implement alternative that could aid in both the diagnosis and the follow-up of GBM patients. Cancer cells release tumoral content into the bloodstream, such as circulating tumor DNA, circulating microRNAs, circulating tumor cells, extracellular vesicles, or circulating nucleosomes: all these could serve as a marker of GBM. In this narrative review, we discuss the current knowledge, the advantages, and the disadvantages of each circulating biomarker so far proposed.

## 1. Introduction

Glioblastoma (GBM) is the most common and malignant primary brain tumor. The cellular origin of GBM is uncertain, but it could arise from neural stem cells or glial precursor cells, which accumulate mutations [1]. GBM account for almost half of malignant Central Nervous System (CNS) tumors and are mainly located in frontal (28.6%), temporal (25%), and parietal (15.3%) lobes [2]. The annual incidence rate is 3.23 per 100,000 population in the United States, with the highest rates between 75 and 84 years [2]. There is no identifiable cause for GBM, but some genetic syndromes predestinate a higher risk [3]. The 2021 World Health Organization (WHO) classification of tumors of the CNS, based on the 2016 updated edition and on the recommendations of the Consortium to Inform Molecular and Practical Approaches to CNS Tumor Taxonomy—Not Official WHO (cIMPACT-NOW), emphasizes not only histological investigations but also the use of molecular markers in classifying brain tumors [4,5,6]. According to this classification, GBM is defined as isocitrate dehydrogenase (IDH)-wildtype diffuse astrocytic glioma, arising in adults and showing one or more of the following features: microvascular proliferation, necrosis, telomerase reverse transcriptase (TERT) promoter mutation, epidermal growth factor receptor (EGFR) gene amplification, combined gain of entire chromosome 7, and loss of entire chromosome 10 [4]. At present, GBM is mainly diagnosed by neuroimaging techniques and by histopathological and molecular analysis of the resected or biopsied tissue. Both imaging and tissue-based methods have, despite their advantages, some important limitations which will be discussed in detail.

Current treatment for newly diagnosed GBM relies on a combination of maximal safe surgical resection, fractionated radiotherapy, and temozolomide (TMZ) -based alkylating chemotherapy [7]. Despite this multimodal treatment, most of the patients (70%) experienced relapse within one year from diagnosis, median survival is only 15 months, and 5-year survival is only 7% [2,8,9]. The standard technique for patients’ follow-up is brain magnetic resonance imaging (MRI). However, the discrimination between actual relapses and the so-called pseudoprogression (PsP), consisting of treatment-related lesions mimicking a recurrence, is frequently challenging and, therefore, changes induced by, e.g., radiation, may be misinterpreted as tumor progression [10].

In this context, liquid biopsy, which involves the detection and quantification of tumoral content released by tumors into the biofluids, has emerged as a promising and non-invasive diagnostic tool complementary to conventional methods [9]. The aim of the present review is to summarize the current knowledge of the most important circulating biomarkers so far proposed for GBM, in particular, circulating tumor DNA (ctDNA), circulating microRNAs (miRNAs), circulating tumor cells (CTCs), extracellular vesicles (EVs), and circulating nucleosomes.

## 2. Pitfalls and Limitations of Current Techniques for GBM Diagnosis and Follow-Up

### 2.1. Neuroimaging

**Figure 1:**
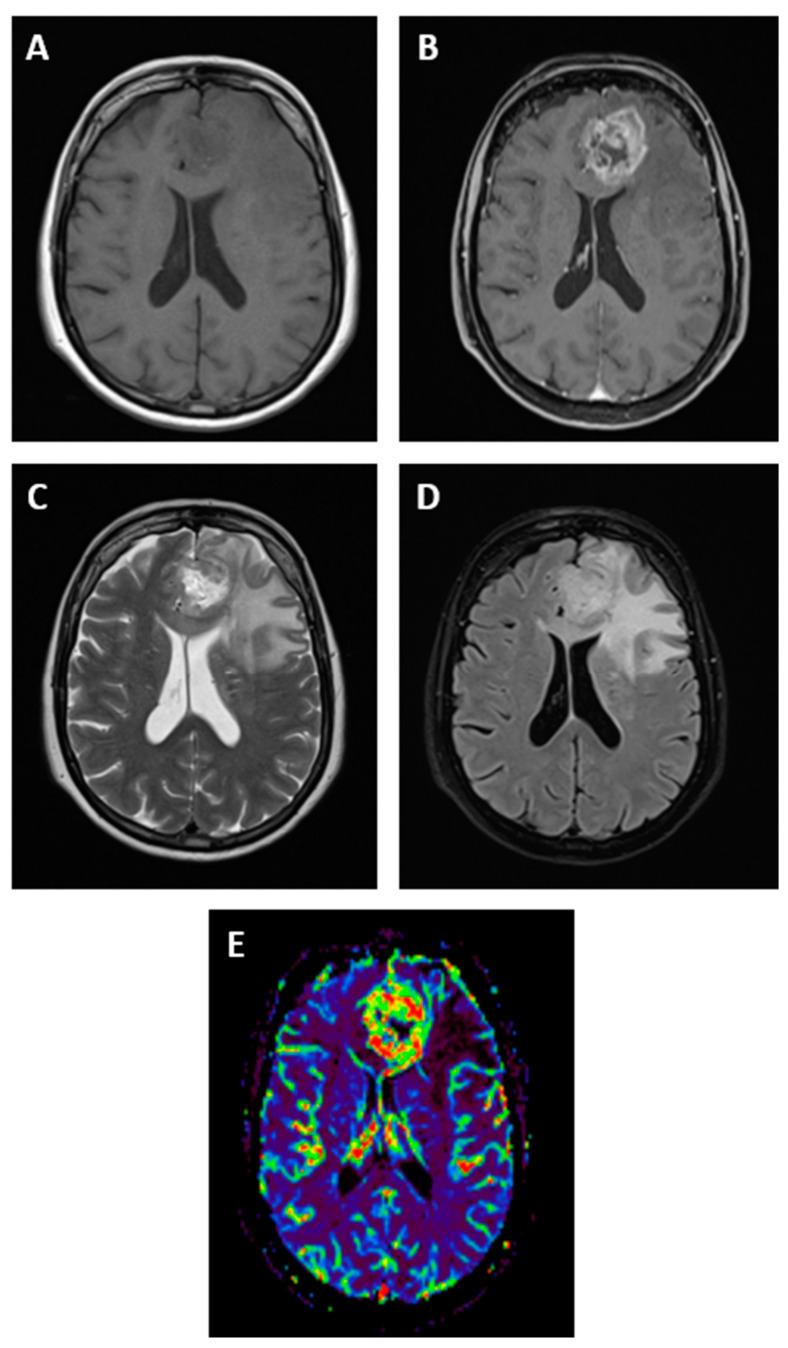
Magnetic resonance imaging features of a patient with GBM in the left frontal lobe. GBM appears as a hypointense or isointense mass on T1-weighted images (**A**), with a ring pattern of enhancement on gadolinium-enhanced images reflecting the increased blood–brain barrier permeability (**B**). GBM is typically hyperintense on both T2-weighted (**C**) and fluid-attenuated inversion recovery (FLAIR, **D**) images, surrounded by vasogenic edema. Perfusion-weighted imaging is an advanced MRI method useful in GBM for differential diagnosis (**E**).

At present, the gold standard for GBM radiological diagnosis is MRI since it overperforms computed tomography (CT) for a number of reasons, such as better anatomical resolution, better capacity to identify GBM features, and the possibility to realize more advanced analyses (i.e., brain tumor spectroscopy) [11,12]. Only in some specific settings, such as MRI not being available, not feasible (i.e., presence of metallic implants), or in urgent situations (i.e., life-threatening brain hemorrhage), brain CT is still used [12]. For this reason, the present review will focus on brain MRI as the technique of choice for the initial diagnosis and follow-up of GBM. Various morphological sequences are used to evaluate tumor lesions on MRI, such as pre- and post-gadolinium contrast-enhanced T1-weighted, T2-weighted, fluid-attenuated inversion recovery (FLAIR), and others, to characterize GBM morphology [13]. In addition to morphological imaging, advanced MRI techniques have more recently emerged, such as perfusion-weighted imaging, magnetic resonance spectroscopy, diffusion-weighted imaging, and its variants, such as diffusion tensor imaging [14] (Figure 1). These advanced techniques can provide more detailed information on tumor properties, and they can be particularly useful in differential diagnosis given their better sensitivity and specificity compared to conventional MRI, which showed pooled sensitivity and specificity of 68% (95% CI 51–81) and 77% (95% CI 45–93), respectively [14,15].

Despite the continuous improvement of MRI-based diagnostic techniques, three main limitations can still be identified.

Firstly, MRI does not always discriminate GBM unequivocally from other tumorous or non-tumorous diseases, or it does not clearly discriminate the tumor mass from other concomitant pathological processes implicated in the disease, such as inflammation, edema, scaring, or bleeding, possibly leading to an overestimation of the extension of the tumor mass [16]. As a consequence, the misinterpretation of MRI images can occur, and GBM is therefore confused with other brain tumors such as low-grade gliomas, brain metastases, or primary CNS lymphoma but also with non-neoplastic pathologies such as brain abscess, demyelinating diseases, the hemorrhagic transformation of ischemic strokes, and others [12,16].

Secondly, the exact correlation between MRI features and GBM’s molecular markers, which have become essential in GBM diagnosis, must still be elucidated. At present, radiogenomics, which studies such correlations, has become an active research field [17]. Connections between some molecular alterations found in GBM such as changes in O^6^-methylguanine-DNA methyltransferase (MGMT), IDH, EGFR, platelet-derived growth factor, vascular endothelial growth factor (VEGF), phosphatase and TENsin homolog, TERT genes, and radiophenotypic manifestations have been shown [17,18]. However, current results are divergent because of the interpatient heterogeneity of GBM, the small size of cohorts that cannot represent this heterogeneity, and the lack of standardized guidelines for systematic image acquisition and analysis [17]. In addition, studies evaluating the link between MRI features and GBM molecular alterations have not been validated prospectively, currently limiting the clinical implementation of radiogenomics to help predict genomic modifications of GBM [18].

Thirdly, the distinction between actual tumor recurrence or progression following treatment and PsP can be challenging in a relatively high number of cases. Radiologically, PsP is defined as a subacute contrast-enhancing lesion that becomes apparent in MRI images within or around the GBM resection cavity [10]. Just like actual recurrences, PsP can be associated with clinical deterioration, or it can be asymptomatic [10]. PsP occurs in 10–30% of GBM patients who undergo their first MRI scan, usually within the first 12 weeks after chemoradiotherapy [10]. It seems to occur more frequently in patients with MGMT promoter methylation (91%) than in patients with unmethylated MGMT promoter (41%) [19]. Overexpression of p53 is also potentially correlated with the development of PsP [20]. Interferon regulatory factor 9 and X-ray repair cross-complementing 1 are highly expressed in patients with PsP [21]. The exact mechanisms underlying PsP are not known. However, the direct destruction of tumor and endothelial cells in the tumor area induced by chemoradiotherapy might lead to inflammation, increased vascular permeability, and edema, resulting in the abnormal contrast enhancement seen in this condition [22]. Furthermore, the expression of hypoxia-related molecules by the tumor and surrounding cells is triggered by treatment-related cellular hypoxia and may play a role in increased endothelial permeability and contrast enhancement in neuroimaging [10]. Contrarily to actual recurrences, PsP resolves spontaneously over time without any change in the current treatment. For obvious reasons, discrimination between an actual recurrence requiring a modification of the therapeutic approach and PsP is of utmost importance for GBM patients [10]. Nevertheless, at present, no biomarker has been clinically validated to better distinguish true progression from PsP [9]. A study by Topkan et al. revealed that, in a series of 28 GBM patients diagnosed with radiological progression at MRI and who were re-operated, 12 (42%) showed pathological features of PsP with no sign of actual recurrence [23]. Advanced MRI techniques could improve the distinction between PsP and true progression. Results of the meta-analysis of Yu et al. revealed that diffusion imaging with apparent diffusion coefficient (ADC) values could discriminate PsP from true progression with higher ADC observed for PsP [24]. The perfusion MRI method could also differentiate PsP from tumor progression with higher relative cerebral blood volume values in true progression compared to treatment effects [25].

Positron emission tomography (PET) is another neuroimaging method that uses radiolabeled molecules to study the biochemical activity of tissues and might provide higher sensitivity compared to MRI in distinguishing between PsP [26]. Many PET tracers can be used; however, in the context of GBM, amino acid analogues are generally preferred, given their relatively low uptake in normal brain and the high uptake in GBM [26]. It has been shown that the amino acid uptake is increased in tumors at progression but not in brain areas of treatment-related changes [26]. For example, ^18^F-fluoroethyltyrosine (^18^F-FET) could facilitate the detection of PsP because ^18^F-FET uptake is significantly lower in patients with PsP than in those with true progression [27].

### 2.2. Tissue Biopsies

Due to the MRI limitations described above, the histopathological and molecular analysis of the biopsied or resected tissue is still considered the gold-standard method to diagnose GBM [9]. In addition to very rare cases, a surgical approach is always necessary for these patients. The typical histopathological features of GBM include increased cellularity, nuclear atypia, microvascular proliferation, and necrosis [28]. As already discussed, in addition to the histopathological analysis, immunohistochemical staining and molecular tests are usually performed to highlight specific genetic and epigenetic features of GBM such as MGMT promoter methylation, IDH-wildtype, TERT promoter mutation, combined gain of entire chromosome 7 and loss of entire chromosome 10, EGFR amplification, and others [9]. Despite being accurate, direct tissue analysis also has some limitations.

Obviously, tissue analysis requires collecting tumor samples by means of a surgical procedure. At diagnosis, obtaining tumor samples normally does not represent an issue since most GBM patients undergo surgical resection. However, a small proportion of patients is not eligible for surgical resection (old age, deep-seated lesions, etc.), and an image-guided stereotactic biopsy could then be performed. Nevertheless, also for such minimally invasive biopsies, a surgical risk exists and includes hemorrhage or brain swelling within and around the tumor with potential permanent deficits or death [29,30]. After the standard of care treatment, GBM patients usually develop MRI alterations suggestive of recurrence within one year. As already stated, 10–30% of these cases can be PsP: tissue analysis via stereotactic biopsies, for cases where surgery is not indicated, could help distinguish PsP for actual recurrence [10]. However, this could further expose the patients to non-negligible surgical risks. Furthermore, given the risk related to brain tumors biopsies, repeated sampling during tumor progression is usually not performed, thereby limiting the possibility of monitoring the treatment response and identifying the emergence of therapeutic resistance early [29].

Another limitation is that the analysis of tumor tissue is restricted, for technical reasons, to a small part of the whole tumor mass. In the case of stereotactic biopsies, the size of the analyzed tissue is even smaller. Given the increasing evidence of the high degree of spatial heterogeneity in GBM, an analysis conducted on a small fragment might not be representative of the whole tumor [31]. Therefore, critical genomic alterations can possibly be missed, and typical histopathological characteristics of the GBM may be underrepresented [18,32].

At last, the tumor also evolves continuously over time due to treatments, clonal evolution, and hypoxia; thus, tissue biopsies capturing only a static snapshot of the entire tumor cannot evaluate the tumor activity in real-time [33].

## 3. Circulating Biomarkers in GBM

**Figure 2:**
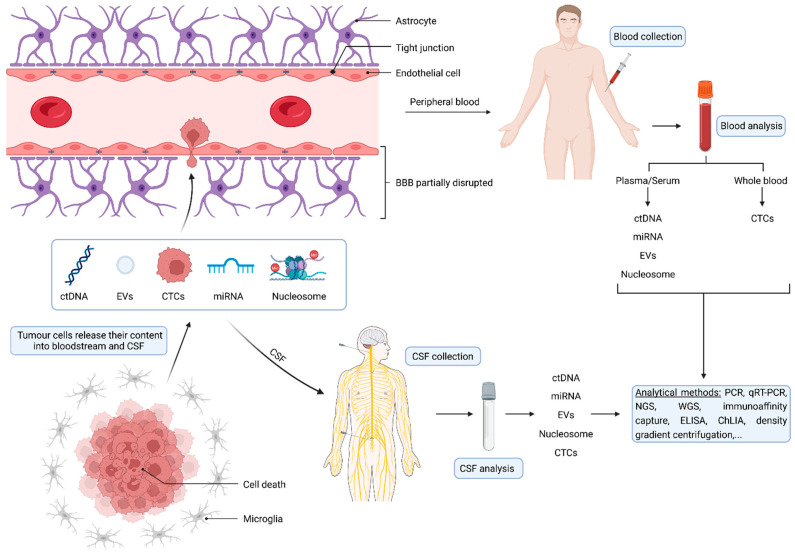
Schematic representation of circulating biomarkers that are disseminated from the tumor into the bloodstream across the partially disrupted blood–brain barrier (BBB). Circulating biomarkers are also released directly into the cerebrospinal fluid (CSF). Next, blood or CSF can be sampled non-invasively and analyzed through different analytical methods. Several classes of biomarkers can be accessed and quantified in liquid biopsies, such as circulating tumor DNA (ctDNA), circulating microRNAs (miRNAs), circulating tumor cells (CTCs), extracellular vesicles (EVs), and circulating nucleosomes.

Given the limitations of MRI and tissue biopsies described above, the identification and validation of alternative and complementary diagnostic techniques helping in the diagnosis, treatment, and follow-up of GBM patients represent an urgent and unmet clinical need. In the following section, we will discuss the biological bases, the advantages, and the disadvantages of all the different types of circulating biomarkers proposed for GBM (Figure 2).

### 3.1. Circulating Tumor DNA

In 1948, Mandel and Metais described for the first time the existence of cell-free nucleic acids, including cell-free DNA (cfDNA), in the blood of healthy individuals and patients with different metabolic or oncological disorders [34]. A higher quantity of cfDNA in the serum of patients with cancer compared to healthy individuals was firstly discovered in 1977 by Leon et al. [35]. Stroun and co-workers reported that neoplastic characteristics were found in the cfDNA of cancer patients [36]. Other studies confirmed that several tumor-related genomic alterations, such as mutations in oncogenes and/or tumor-suppressor genes [37], epigenetic aberrations [38], and microsatellite instability [39], were present in the cfDNA. cfDNA released by tumor cells in the bloodstream and carrying genetic and/or epigenetic alterations of the original tumor is called circulating tumor DNA (ctDNA) [40]. Interestingly, several studies showed that ctDNA is highly specific to the original tumor, and a good concordance has been shown between the mutational profile of ctDNA and matched tumor tissue from different cancers [41,42,43]. The mechanisms of release of circulating ctDNA into the blood are not fully elucidated yet [44]. The apoptosis of neoplastic cells (e.g., due to hypoxia) is a source of DNA fragments which have, in that case, a length of about 130–180 base pairs, reflecting the action of a caspase-activated DNase that degrades chromatin into mono- and oligonucleosomes [33,45]. Necrosis of tumor cells is another proposed mechanism to explain the release of ctDNA into body fluids, but ctDNA from necrosis is typically of larger size than ctDNA from apoptotic cell death [45]. Macrophages can also release DNA fragments after the engulfment of necrotic cancer cells [46]. Fragments released by normal cells are typically cleared by phagocytosis, and the background level of cfDNA is generally low in the circulation of healthy individuals, with an average concentration of 30 ng/mL [47,48]. In patients with cancer, clearance mechanisms are overwhelmed by the DNA fragments released from tumor cells and thus a proportion of cfDNA (as little as 0.01% or up to 90%) present in the circulation is composed of ctDNA [47]. There are two main methods to detect mutations in cfDNA, thereby identifying ctDNA. Polymerase chain reaction-based techniques target known point mutations, and next-generation sequencing or whole genome sequencing allows for the detection of novel and unknown mutations [49]. Of note, the background level of cfDNA is higher in serum compared to plasma, probably because of contamination with DNA released from immune cells lysed during the clotting process; therefore, plasma samples are preferred for the study of ctDNA [50].

The studies aimed at identifying ctDNA in GBM are summarized in Table 1. The number of patients included in each study was relatively low, especially when cerebrospinal fluid (CSF) was used because of the invasive procedure required for its collection. Nevertheless, it seems that the detection rate of ctDNA is higher in CSF compared to plasma and serum. One possible reason is that, even if partially disrupted, the blood–brain barrier (BBB) still limits the passage of ctDNA from the primary brain tumor to blood circulation [51]. Other reasons could be the lower distance that ctDNA has to travel (in the absence of anatomical filters) before being sampled, the less efficient ctDNA clearance mechanisms, and the lower levels of background cfDNA in CSF than in blood [9,52]. Despite promising results, the use of ctDNA as a biomarker, in particular for GBM, is challenging. Firstly, the quantity of ctDNA varies with the originating tissue type and the stage of cancer, with higher amounts observed in advanced-stage cancers, while the technique has a potential mostly in early stage diagnosis [53]. Furthermore, gliomas have been shown to be among the tumor types with the lowest level of detectable ctDNA [53]. Secondly, ctDNA has a short half-life (less than two hours) which requires fast processing after sampling [54]. Thirdly, even if detectable, the concentration of ctDNA in the blood is very low in cancer (180 ng/mL), probably even less in the case of GBM, thus requiring high-sensitive techniques for its identification and differentiation from normal cfDNA [48].

### 3.2. Circulating microRNAs

The existence of miRNAs was first described in 1993 in Caenorhabditis elegans [66], and they are the most common small RNAs with a size of approximately 21–23 nucleotides [67]. They are single-stranded and non-coding RNAs that can regulate up to 30% of the protein-coding genes in the genome [67]. Generally, miRNAs downregulate gene expression at the post-transcriptional level by binding to their messenger RNA (mRNA), leading to the translational inhibition or degradation of the target mRNA [67]. They play a role in physiological and pathological processes, such as cancer. miRNAs can be present in the blood and CSF of GBM patients as circulating cell-free nucleic acids or can even be captured within extracellular vesicles (EVs), providing higher stability [9]. Some studies reported circulating miRNAs dysregulation in glioma patients (Table 2).

In addition to the differentiation of GBM patients from healthy individuals (Table 2), these studies reported that the altered expression of some miRNAs (downregulation: miR-128, miR-342-3p, miR-16, miR-497, miR-125b, miR-205; upregulation: miR-210, miR-454-3p, miR-182, miR-20a-5p, miR-106a-5p, miR-181b-5p), could discriminate between patients with GBM and those with lower-grade gliomas [69,71,72,73,74,75,76,77,79]. The sensitivities and specificities reported for these circulating miRNAs range from 58% to 99% and 67% to 100%, respectively [68,69,70,71,72,73,74,75,76,77,78]. miRNAs expression could also differentiate GBM from other brain pathologies. Sun et al. reported lower serum miR-128 expression in glioma patients, including GBM, than in patients with meningioma [71]. D’Urso et al. discovered that miR-16 was downregulated in GBM patients compared to patients affected by different neurological diseases [72]. Yue et al. showed that levels of miR-205 were significantly lower in patients with gliomas than those with other brain tumors such as meningioma, primary CNS lymphoma, and pituitary adenoma [77]. However, Wang et al. found that the levels of three miRNAs (miR-21, miR-128 and miR-342-3p) were not significantly different between patients with GBM and those with other brain tumors such as meningioma or pituitary adenoma [69]. It was also reported that the miRNAs expression varied during the disease course, such as before and after treatment or at recurrence. Therefore, the variation of miRNAs expression could aid in GBM follow-up but is not specific for its diagnosis as it is also detected in other brain tumors. Wang et al. found that the plasma levels of three miRNAs, miR-21, miR-128, and miR-342-3p, which were dysregulated before operation, almost returned to normal levels after surgery and chemoradiotherapy [69]. Yue et al. reported that miR-205 expression was significantly lower when the tumor relapsed compared to right after surgery [77]. Swellam et al. found that miR-221 and miR-222 expression decreased after treatment compared with pre-treatment levels [78]. Morokoff et al. discovered that dynamic changes of miR-320e correlated with tumor volume assessed by MRI in GBM patients. Interestingly, this miRNA could also help to discriminate PsP observed in MRI from true progression [80]. Siegal et al. reported that levels of miR-10b and miR-21 in patients with high-grade gliomas, including GBM, tended to be higher during treatment with bevacizumab but further investigations were needed to confirm their role as biomarker for tumor response [81].

These clinical data suggest that the dysregulation of miRNAs expression, upregulation or downregulation, can be used as a circulating biomarker for diagnosing or monitoring GBM. In the former case, the identification of miRNAs which are specific to GBM is mandatory to avoid misclassification. Nevertheless, even if non-specific compared to other tumor types, circulating miRNAs could still serve as a non-invasive technique for screening patients with suspicion of GBM. However, although these results are interesting, some limitations exist, such as the small size of cohorts and the lack of standardized methods for blood collection, RNA extraction, and sequencing. miRNAs are also generally less specific compared to ctDNA [52].

### 3.3. Circulating Tumor Cells

The presence of CTCs was firstly described in cancer patients in 1869 [82]. CTCs are cells that are shed into the circulation and originate from either the primary tumor or metastatic deposits [83]. CTCs undergo an epithelial-mesenchymal transition that increases their migratory potential and allows them to intravasate into the circulation; therefore, CTCs are important for the development of metastases [84]. They can be released as single cells or as clusters which have higher survival and metastatic potential compared to single CTCs [85]. There are some technological challenges in the isolation and characterization of CTCs due to their scarcity in the blood (usually, 1 CTC in 10^9^ normal blood cells or 1–10 cells per 10mL of blood) [29,86]. CTCs can be enriched based on biological or physical properties or a combination of both [86]. Protein expression-based technologies are biological isolation methods that are divided into positive and negative selection. The first one captures CTCs by targeting, with antibodies, tumor-specific markers that are expressed by CTCs and not by normal blood cells [86]. Positive selection of CTCs that highly express epithelial cell adhesion molecule (EpCAM) marker is commonly used to isolate CTCs from proliferating carcinomas [52]. However, CTCs from GBM do not express EpCAM due to a more mesenchymal phenotype adopted by these tumor cells [9]. Consequently, other isolation methods of CTCs are required in patients with GBM. Negative selection relies on the enrichment of CTCs by removing the unwanted leukocytes of whole blood using antibodies against leukocyte antigens [86]. CTCs can also be enriched based on their physical properties, which are different compared to normal blood cells. Various devices based on cell filtration, centrifugal force, or dielectrophoresis have been developed [86]. Metastases of GBM outside of the brain rarely occur. Indeed, only 0.4–0.5% of GBM patients present with metastases in sites such as the liver, lungs, lymph nodes, and bones [87]. Some reasons may explain the low rates of extracranial metastases, including the low and short survival of patients with GBM, suppression of tumor cell growth outside the brain by the immune system, and the presence of the BBB, which prevents the GBM cells from intravasate into the circulation [9,88].

Despite the rarity of metastatic spread in GBM, some studies reported the presence of CTCs in GBM patients (Table 3). However, the number of studies focusing on CTCs detection in GBM patients is low as well as the number of patient samples in each study [87,89,90,91,92]. Moreover, it is difficult to compare the results given a lack of a standard method for isolation and characterization of CTCs. Nevertheless, interesting clinical data have been reported. MacArthur et al. found markedly decreased numbers of CTCs after radiotherapy in GBM patients, while an increased level of CTCs was reported in a single patient who has tumor recurrence, suggesting a potential role of CTCs in monitoring disease course [89]. Sullivan et al. highlighted both a greater frequency of CTCs in patients with progressive disease and overexpression of the mesenchymal genes in CTCs, which increased their migratory potential as mentioned above [90]. In conclusion, there are two main difficulties with CTCs. They are rare in the blood and therefore difficult to detect with current techniques. The other reason is the lack of standardized methods to isolate and characterize CTCs. Nevertheless, CTCs from GBM could be of interest for molecular diagnosis, prognosis, monitoring during the disease course, as well as following the onset of therapy resistance, but further investigations are required.

### 3.4. Extracellular Vesicles

EVs are small membrane-bound spheres released by normal and tumor cells [93]. There are two main categories of EVs, which differ primarily in their origin and size. Microvesicles (MVs) are formed directly from the budding of the cell membrane and have a size between 50 and 500 nm (even up to 1 µm) [94]. By contrast, exosomes are smaller; that is, a diameter between 50 and 150 nm and originate from the endosomal system [94]. Indeed, exosomes are intraluminal vesicles that are formed by the invagination of the endosomal membrane during the maturation of multivesicular endosomes (MVEs). Then, the fusion of MVEs with the cell membrane leads to the release of exosomes in the extracellular environment [94]. An important advantage of EVs compared with free nucleic acids and CTCs is that the biomolecules within EVs are protected from enzyme degradation in the extracellular medium through the surrounding lipid bilayer, which also allows them to cross an intact BBB [95]. Cargo of EVs can contain various molecules such as mRNAs, miRNAs, proteins, or lipids specific to the cell of origin [96]. Alongside direct cell–cell contact or secretion of molecules such as hormones, neurotransmitters, or cytokines, EVs play an important role in intercellular communication as their released cargo can be captured by other surrounding or distant cells, altering the phenotype of these cells [97]. Therefore, the phenotype of recipient cells is influenced by the genetic information and proteins transferred by Evs [9]. For example, a study showed that exosomes released from hypoxic GBM cells overexpress VEGF-A which is taken up by brain endothelial cells, resulting in BBB disruption due to the downregulation of claudin-5 and occludin [98]. Different methods to isolate EVs are used, including differential centrifugation gradients and immunoaffinity capture [99]. Koch et al. analyzed blood samples at different treatment times from 11 patients with GBM and 7 healthy individuals. They showed that the number of MVs was significantly lower in patients with stable disease or PsP compared to patients who underwent true tumor progression [100]. Evans et al. demonstrated that an increase in MVs count during chemoradiotherapy was associated with poor overall survival and earlier disease recurrence [101]. Skog et al. identified EGFRvIII, a specific mutation of GBM, in MVs isolated from the sera of seven patients out of the 25 patients with GBM. In contrast, EGFRvIII was not detected in MVs isolated from the sera of healthy controls. The authors also highlighted that the released cargo of MVs, including angiogenic proteins in addition to EGFRvIII, promoted the angiogenic phenotype of normal brain endothelial cells and the proliferation of glioma cells [102]. Osti et al. reported an increased concentration of plasma EVs in GBM patients at diagnosis in comparison with healthy individuals and patients with other brain diseases. The level of plasma EVs significantly decreased in GBM patients after surgical resection of the primary tumor, returning to a level similar to that of healthy subjects. Interestingly, the concentration of EVs increased again in patients facing disease recurrence [103]. André-Grégoire et al. demonstrated higher EVs level in GBM patients compared to heathy controls. They also showed that EVs emanating from GBM stem cells were enriched with cargoes dedicated to cell adhesion after TMZ treatment, suggesting that TMZ could promote the release of molecules favoring tumor progression [104]. Chandran et al. identified syndecan-1 as a plasma EV constituent that could be used to distinguish GBM from low-grade gliomas [105]. Lan et al. reported higher serum exosomal miR-301a expression levels in patients with high-grade gliomas, including GBM, compared to healthy controls. miR-301a levels significantly decreased after surgery and increased again when the tumor relapsed, suggesting that miR-301a might be derived from exosomes secreted by tumor cells [106]. Ebrahimkhani et al. selected a panel of seven exosomal miRNAs, miR-182-5p, miR-328-3p, miR-339-5p, miR-340-5p, miR- 485-3p, miR-486-5p, and miR-543, to discriminate GBM patients from healthy individuals with an accuracy of 91.7% [107]. Manterola et al. found that a signature of two serum exosomal miRNAs, miR-320 and miR-574-3p, as well as a small noncoding RNA, RNU6-1, were upregulated in 75 GBM patients compared to healthy subjects [108]. Santangelo et al. reported that three miRNAs, miR-21, miR-222 and miR-124-3p, were increased in serum exosomes of patients with GBM in comparison with healthy individuals but markedly decreased after surgical resection [109]. All these clinical data suggest a potential role of EVs for the diagnosis, monitoring, and prognosis of GBM. However, the cohorts’ size is again small; therefore, larger cohorts are needed to clinically validate the potential roles of EVs. In addition, the lack of standardized methods to isolate them is another limitation for the clinical utility of EVs and true specificity studies are needed to see whether these EVs are able to differentiate GBM from other (brain) cancers.

### 3.5. Circulating Nucleosome-Associated Histone Modifications

Chromatin structure was described in 1974 by Kornberg [110]. It consists of repeated units of nucleosomes that are formed by an octamer composed of two copies, each of four core histone proteins (H2A, H2B, H3 and H4), which are highly conserved between species [111]. A total of 146-147 base pairs of DNA are wrapped almost twice around this octamer. Nucleosomes are linked together by linker DNA which is associated with proteins, including a histone protein named histone H1 [111]. Each histone protein has a relatively globular form, allowing histone–histone interactions, with an amino-terminal tail (20–35 amino acid residues) rich in positively charged basic residues [112]. This tail protrudes from the surface of the octamer and is subject to histone post-translational modifications (PTMs). Only H2A protein contains an additional ~37 amino acid carboxy-terminal tail that extends from the surface of the nucleosome [112]. The nucleosome is the core unit of the chromatin structure and plays an important role in the main nuclear functions such as DNA transcription, replication, or repair [113]. Histone PTMs regulate these nuclear functions, especially the regulation of genes, in the same way as DNA methylation, which is another mechanism of epigenetic regulation. These PTMs participate in the development and progression of cancer by enhancing the expression of oncogenes and/or silencing tumor-suppressor genes [113]. Various PTMs exist, with the most common ones being methylation, acetylation, phosphorylation, ubiquitination, or sumoylation. Other PTMs have recently been discovered, including glycosylation, homocysteinylation, and crotonylation [114]. These PTMs, mainly the methylation, can exist in different degrees (mono-, di- or tri-) at a single amino acid [114]. Different enzymes influence PTMs, “writers” that add modifications on histones such as histone acetyltransferases, “erasers” which remove PTMs such as histone deacetylases and “readers” that bind to PTMs marks through PTM-specific binding domains such as 53BP1 [114]. Acetylation is usually linked to gene transcription as the addition of an acetyl group to a lysine neutralizes the positive charge of this amino acid. This causes a decreased electrostatic interaction between the lysine residue and negatively charged DNA resulting in more “open” chromatin, thus accessible for transcription factors [114]. Methylation is associated with gene expression or repression depending on the amino acid methylated and the degree of methylation [113]. Different PTMs present on histone tails act together to define a “histone code” that can be read by cellular proteins resulting in various cellular processes such as transcription activation or repression, DNA replication, or repair [113]. Variants of the core histones are called histone variants, and their PTMs make the “histone code” more complex as their incorporation also influences different chromatin-templated processes [114]. Nucleosomes are stable structures in circulation with an annual decrease of nucleosome concentration of about 7% in each stored sample at −70 °C [115]. Nucleosomes and their PTMs can be detected, for example, by using immunoassays such as the enzyme-linked immunosorbent assay (ELISA) or chemiluminescence immunoassay (ChLIA) in plasma and serum [114]. ELISA and ChLIA are simple methods commonly used for detecting a specific biomarker in a complex matrix, and they can be implemented on automated platforms allowing faster and more reproducible results.

In cancer, including GBM, cell death leads to the release of nucleosomes into the bloodstream, which are carried mainly as mononucleosomes or oligonucleosomes with ctDNA [114]. A higher quantity of nucleosomes is detected in patients with cancer due to increased cellular turnover compared to healthy individuals and the cytotoxic effect of treatments that also leads to cell death and the release of nucleosomes [114]. However, the elevated level of nucleosomes is not specific to cancer and GBM per se. High levels of nucleosomes are also observed in non-neoplastic diseases such as stroke, trauma, and sepsis, limiting the clinical use of the overall level of nucleosomes as a unique biomarker for cancer detection due to the known lack of specificity [114]. Recent studies reported that PTMs present on circulating nucleosomes could be more specific than the overall rate of nucleosomes and thus could be explored as biomarkers. For example, H3K9me3 and H4K20me3 have been associated with colorectal cancer, and their levels were significantly decreased in the plasma of patients with colorectal cancer compared to healthy subjects [114]. Rahier et al. found that a signature of three histone modifications, in addition to the overall level of circulating nucleosomes, could discriminate patients with colorectal cancer from healthy individuals [116].

Nevertheless, in GBM, PTMs of nucleosomes are not fully defined, and so a specific signature for GBM has not yet been identified. However, enzymes that regulate PTMs can be dysregulated in GBM. It has been reported that the overexpression of several histone deacetylases and demethylases in GBM, such as lysine-specific histone demethylase 1, may influence the epigenetic status of brain cells and, therefore, the expression of genes implicated in the development or the progression of cancer [117,118]. Moreover, several histone mutations are commonly observed in pediatric high-grade gliomas [117]. The H3K27M mutation, a lysine-to-methionine substitution occurring at position 27 in either H3.1 or H3.3 histone genes, is often detected in children with diffuse intrinsic pontine glioma [119]. This mutation blocks the lysine methyltransferase activity of the polycomb repressive complex 2, resulting in a global loss of H3K27 methylation and altered gene expression [117]. H3K27M mutation has also been identified in adults with a glioma [119]. The H3G34R/V mutations are substitutions of glycine 34 to arginine or valine in the H3.3 histone gene and are observed in children or adolescents with gliomas located on the cerebral hemispheres [119]. These mutations lead to the methylation redistribution of the activation H3K36 mark, resulting in transcriptional dysregulation [117]. Other epigenetic marks are dysregulated in GBM, such as methylation of histone 3 lysine 4 (H3K4), which is decreased in severe GBM cases, leading to gene repression [118]. H3K18Ac, acetylation of histone 3 at lysine 18, is one of the most frequent PTMs, and an altered expression of H3K18Ac has also been observed in various cancers, including GBM for which low levels are associated with a better prognosis for patients [120].

Thus, it would be of interest to define a profile of PTMs present on nucleosomes of GBM patients, which may, in addition to clinical symptoms and non-invasive MRI, guide the diagnosis and the follow-up of GBM patients. Further research is required to define the benefit of such a non-invasive and easily accessible method for complementing the diagnosis of GBM and ensuring the monitoring of tumor progression.

## 4. Conclusions and Discussion

As the mortality rate of GBM is high, there is an urgent need to develop, if possible, minimally invasive methods for the early detection and monitoring of GBM patients. Currently, MRI is used for the radiological diagnosis of GBM. Although this non-invasive method allows anatomic visualization of the brain tumor, it does not differentiate GBM from concomitant pathological processes and other brain diseases and does not discriminate true progression from PsP, a major issue in clinical practice. Advanced MRI and amino acid PET can be helpful in this situation. In addition, the correlation between MRI features and GBM’s molecular alterations still has to be elucidated. Therefore, tissue biopsy is required for a complete diagnosis of GBM. However, tissue biopsies also have several limitations. First, there is a surgical risk which restricts repeated sampling. Additionally, they might not be representative of the whole tumor. In addition, they cannot evaluate the tumor activity in real-time. To overcome these limitations, the analysis of circulating biomarkers on liquid biopsy emerges as an alternative or complementary method to conventional techniques to help in GBM diagnosis and monitoring. Liquid biopsy offers some advantages, such as the non-invasive feature and easiness of the procedure. It also allows multiple samplings during the course of disease and treatments. Individually, each type of circulating biomarker has some advantages and disadvantages that are summarized in Table 4, highlighting that a combination of biomarkers could be more clinically useful compared with a unique biomarker. This could improve diagnostic sensitivity and specificity, an actual important drawback of current GBM liquid biomarkers.

Circulating biomarkers can be sampled from different biofluids such as blood and cerebrospinal fluid. Due to the invasiveness of the procedure to collect cerebrospinal fluid, a lumbar or cisternal puncture is required, blood is the optimal biofluid for liquid biopsy. However, some regions of the tumor are protected by an intact BBB that prevents the passage of circulating biomarkers into the bloodstream resulting in low levels of them in the blood of patients compared to levels in the CSF. Ultra-sensitive analytical methods are therefore needed to improve the applicability and utility of liquid biopsy for detecting initial and relapsed GBM. Recently, single-molecular array (Simoa), described by Rissin et al. in 2010 [121], emerged as a new technology allowing for the detection of molecules at femtomolar concentrations. It paves the way for the quantification of biomarkers present at very low concentrations in the blood. It is especially appropriate for nucleosome modifications, which is particularly interesting because epigenetics arises as a new intensive research area in cancer compared to traditional genetics. Nevertheless, the key point for these circulating biomarkers will result in their capacity to discriminate between (brain) cancer types, as many of these biomarkers are not entirely specific to GBM but are also observed in other cancer types. Thus, it will be mandatory in the future to ensure that these biomarkers are specific, at least in patients sharing similar symptoms at entry, to allow appropriate use of these technologies in the clinical setting.

Additionally, despite the interesting clinical data mentioned in this review, a standardization of the methods is needed as well as more clinical studies and larger cohorts to permit the implementation of these biomarkers in clinical applications.

## Figures and Tables

**Table 1:** Studies reporting ctDNA in GBM. Only studies in which data for GBM patients were available are reported.

Study	Patients (n)	Biofluid	Method	ctDNA Detection Rate
Schwaederle et al. [55]	33	Plasma	NGS	27%
Piccioni et al. [56]	222	Plasma	NGS	55%
Zill et al. [57]	107	Plasma	NGS	51%
Bagley et al. [58]	20	Plasma	NGS	55%
Cordova et al. [59]	13	Plasma	ddPCR	46%
Wang et al. [60]	19	Serum, CSF	Methylation specific PCR assay	37% (Serum), 61% (CSF)
Juratli et al. [61]	38	Plasma, CSF	Nested PCR	8% (Plasma), 92% (CSF)
Wang et al. [62]	11	CSF	WGS	100%
Mouliere et al. [63]	10	CSF	WGS	50%
Martínez-Ricarte et al. [64]	9	CSF	ddPCR	100%
Miller et al. [65]	46	CSF	NGS	59%

Abbreviations: CSF, cerebrospinal fluid; ctDNA, circulating tumor DNA; (dd)PCR, (droplet digital) polymerase chain reaction; NGS, next-generation sequencing; WGS, whole genome sequencing.

**Table 2:** Studies reporting miRNAs in GBM. Only studies in which data for GBM patients were available are reported.

Study	No. of Patients (Cases/Controls)	Controls Type	Biofluid	miRNA	Upregulation or Downregulation	Method
Roth et al. [68]	20/20	Healthy	Blood	miR-128 miR-342-3p	Upregulation Downregulation	qRT-PCR
Wang et al. [69]	10/10	Healthy	Plasma	miR-21 miR-128 miR-342-3p	Upregulation Downregulation Downregulation	qRT-PCR
Yang et al. [70]	33/80	Healthy	Serum	miR-15b, miR-23a, miR-133a, miR-150, miR-197, miR-497 and miR-548b-5p	Downregulation	qRT-PCR
Sun et al. [71]	61/53	Healthy	Serum	miR-128	Downregulation	qRT-PCR
D’Urso et al. [72]	16/30	Neurologic disorders	Serum	miR-16	Downregulation	qRT-PCR
Lai et al. [73]	42/50	Healthy	Serum	miR-210	Upregulation	qRT-PCR
Shao et al. [74]	22/70	Healthy	Plasma	miR-454-3p	Upregulation	qRT-PCR
Regazzo et al. [75]	10/15	Healthy	Serum	miR-497	Downregulation	qRT-PCR
Xiao et al. [76]	39/54	Healthy	Plasma	miR-182	Upregulation	qRT-PCR
Yue et al. [77]	27/45	Healthy	Serum	miR-205	Downregulation	qRT-PCR
Swellam et al. [78]	20/20	Healthy	Serum	miR-221 and miR-222	Upregulation	qRT-PCR

Abbreviations: miR, microRNA; qRT-PCR, quantitative reverse transcription polymerase chain reaction.

**Table 3:** Studies reporting CTCs in GBM.

Study	Patients (n)	Biofluid	Method	CTCs Detection Rate
Müller et al. [87]	141	Peripheral blood	Density gradient centrifugation followed by immunostaining for GFAP	21%
MacArthur et al. [89]	11	Peripheral blood	Density gradient centrifugation followed by telomerase-based test	72% preradiotherapy 8% postradiotherapy
Sullivan et al. [90]	33	Peripheral blood	CTC–iCHIP technology; characterization using antibodies cocktail	39%
Gao et al. [91]	11	Peripheral blood	Examination for aneuploidy of chromosome 8 by FISH	82%
Krol et al. [92]	13	Peripheral blood	Parsortix microfluidic technology; characterization using antibodies cocktail	54%

Abbreviations: CSF, cerebrospinal fluid; ctDNA, circulating tumor DNA; (dd)PCR, (droplet digital) polymerase chain reaction; NGS, next-generation sequencing; WGS, whole genome sequencing.

**Table 4:** Summary of advantages and disadvantages of each diagnostic method.

Diagnostic Method	Advantages	Disadvantages
MRI	Allows initial diagnosis and anatomic characterization of GBM with non-invasive procedure	Difficulty in discriminating GBM from other brain diseases and other concomitant pathological processes Difficulty in correlating MRI features with molecular features Difficulty in distinct actual tumor recurrence from PsP
Tissue biopsy	Allows histologic and molecular characterization of GBM	Highly invasive procedure with risks, limiting repeated sampling May not reflect the intra-tumoral heterogeneity Cannot evaluate the tumor activity in real-time
ctDNA	Higher levels than CTCs Very specific Quantity correlates with the disease stage Easier to collect than CTCs and established detection techniques	Short half-life (<2 h) Released mainly by apoptotic or necrotic cells and therefore represents only a subpopulation of tumor cells Sensitivity of detection limited
miRNAs	Relatively stable	No standardized methods for RNA extraction and sequencing Less specific than ctDNA
CTCs	Highly specific Can provide information on protein, DNA and RNA levels	Lack of standardized methods to isolate and characterize CTCs Low presence in blood May not represent the whole tumor
EVs	Can carry RNAs, proteins, and lipids which are protected from enzyme degradation Can cross an intact BBB Released by all cells, including cancer cells	Lack of standardized methods to isolate EVs released by non-neoplastic cells, so there is a background of nontumoral EVs in the blood
Circulating nucleosome-associated histone modifications	Highly stable Simple methods (ELISA, ChLIA) to detect them Epigenetics is a new intensive field of research	Low specificity

Abbreviations: BBB, blood–brain barrier; ChLIA, chemiluminescence immunoassay; CTCs, circulating tumor cells; ctDNA, circulating tumor DNA; ELISA, enzyme-linked immunosorbent assay; EVs, extracellular vesicles; GBM, glioblastoma; miRNAs; microRNAs; MRI, magnetic resonance imaging; PsP, pseudoprogression.
